# Progressive retinal degeneration of rods and cones in a Bardet-Biedl syndrome type 10 mouse model

**DOI:** 10.1242/dmm.049473

**Published:** 2022-09-20

**Authors:** Sara K. Mayer, Jacintha Thomas, Megan Helms, Aishwarya Kothapalli, Ioana Cherascu, Adisa Salesevic, Elliot Stalter, Kai Wang, Poppy Datta, Charles Searby, Seongjin Seo, Ying Hsu, Sajag Bhattarai, Val C. Sheffield, Arlene V. Drack

**Affiliations:** ^1^ Interdisciplinary Graduate Program in Genetics, University of Iowa, Iowa City, IA 52242, USA; ^2^ Department of Ophthalmology and Visual Sciences, Institute for Vision Research, University of Iowa, Iowa City, IA 52242, USA; ^3^ Department of Biostatistics, University of Iowa, Iowa City, IA 52242, USA; ^4^ Department of Pediatrics, University of Iowa, Iowa City, IA 52242, USA

**Keywords:** BBS10, Bardet-Biedl syndrome, Genetic model, Mouse model, Retinal degeneration

## Abstract

Bardet-Biedl syndrome (BBS) is a multi-organ autosomal-recessive disorder caused by mutations in at least 22 different genes. A constant feature is early-onset retinal degeneration leading to blindness. Among the most common forms is BBS type 10 (BBS10), which is caused by mutations in a gene encoding a chaperonin-like protein. To aid in developing treatments, we phenotyped a *Bbs10* knockout (*Bbs10^−/−^*) mouse model. Analysis by optical coherence tomography (OCT), electroretinography (ERG) and a visually guided swim assay (VGSA) revealed a progressive degeneration (from P19 to 8 months of age) of the outer nuclear layer that is visible by OCT and histology. Cone ERG was absent from at least P30, at which time rod ERG was reduced to 74.4% of control levels; at 8 months, rod ERG was 2.3% of that of controls. VGSA demonstrated loss of functional vision at 9 months. These phenotypes progressed more rapidly than retinal degeneration in the *Bbs1^M390R/M390R^* knock-in mouse. This study defines endpoints for preclinical trials that can be utilized to detect a treatment effect in the *Bbs10^−/−^* mouse and extrapolated to human clinical trials.

## Teaser

**Summary:** A mouse model of Bardet-Biedl syndrome type 10 recapitulates the human disease phenotype and can be used to develop therapies for future clinical trials.

## INTRODUCTION

Bardet-Biedl syndrome (BBS) is an autosomal-recessive disorder caused by mutations in at least 22 different genes that are important for proper function of primary cilia (Mardy et al., 2021; Weihbrecht et al., 2017; Zhou et al., 2022). Retinal degeneration leading to legal blindness is a constant feature. Vision loss is typically associated with some combination of kidney failure, obesity, developmental anomalies, cardiac issues and diabetes; however, it has also been reported as an isolated feature (Forsythe and Beales, 2013; Green et al., 1989; Schachat and Maumenee, 1982). Retinal degeneration usually begins in the first decade of life and results in rapidly deteriorating visual acuity, decreases in the visual field and, eventually, blindness (Green et al., 1989; Schachat and Maumenee, 1982). BBS type 10 (BBS10) is a common form. It is caused by mutations in the *BBS10* gene, which encodes a chaperonin-like protein that helps to assemble the BBSome, a protein complex responsible for ciliary transport (Singh et al., 2020; Stoetzel et al., 2006). BBS10 represents almost 25% of all BBS cases (Forsythe and Beales, 2013) and is thus a high-yield target for treatment. Recently, it was reported that, in BBS10, the onset and progression of retinal degeneration occur earlier and faster, respectively, than in BBS type 1 (BBS1)*,* another common subtype (Davis et al., 2007; Grudzinska Pechhacker et al., 2021). Research identifying points in the course of BBS10 at which treatment interventions are effective will be important for helping the affected population.

BBS mouse models have been developed for many of the genetic subtypes of BBS, to better study the etiology and potential treatment of BBS-associated retinal degeneration (Bentley-Ford et al., 2021; Cognard et al., 2015; Davis et al., 2007; Hsu et al., 2017; Zhang et al., 2011). Each BBS mouse model that has been reported has a retinal phenotype similar to that seen in human patients (Bales et al., 2020; Cognard et al., 2015; Datta et al., 2015; Davis et al., 2007; Nishimura et al., 2004; Zhang et al., 2011, 2013). A previous report utilized a Cre/Lox system, in which the LoxP sites were engineered around exon 2 of the *Bbs10* gene (Cognard et al., 2015). A total knockout was created by breeding these mice with a Cadh16-CreDeleter line. That study explored the retinal phenotype at 2[Supplementary-material sup1]months of age and found that the retinal layers of these mice were unusually thin and that phototransduction was inefficient. However, it did not explore whether the retinal degeneration is progressive or stable, explore differences between rod and cone function, or test functional vision. An electroretinogram (ERG) is a measure of electrical activity and is useful to assess numbers of viable photoreceptors, but it does not correlate with visual acuity or use of vision in the environment. Extensive characterization of a knockout model is necessary to understand the potential for treatment in translational studies.

Here, we report on the details of the retinal degeneration phenotype of a *Bbs10^−/−^* mouse. Serial data on retinal anatomy [histology and optical coherence tomography (OCT)], retinal electrophysiology (ERGs) and functional vision [visually guided swim assay (VGSA)] were collected. The VGSA is modified from similar assays reported in mice (Laird et al., 2019; Pang et al., 2008, 2006; Prusky et al., 2000; Sarria et al., 2015) and is designed to mimic the human multiluminance mobility test (MLMT) (Chung et al., 2018), which is utilized as a functional endpoint in clinical trials of human retinal gene therapies. In the *Bbs10^−/−^* mouse, cone electrical activity is absent from the earliest age tested [postnatal day (P)30]; although rod function is present initially, it is less robust than in controls and diminishes further over time. This differs from most types of retinitis pigmentosa (RP), in which rod loss precedes cone loss. The *Bbs10^−/−^* mouse recapitulates the ‘cone-simultaneous-with-rod or cone-before-rod’ retinal phenotype reported in some BBS10 patients (Grudzinska Pechhacker et al., 2021). A functional assay of cone vision, the light-adapted VGSA, demonstrated that visually guided navigation is possible even when cone electrical function is very low. Comparison to *Bbs1^M390R/M390R^*, a mouse model of BBS1 (Davis et al., 2007), demonstrated that the retinal degeneration in the BBS10 mouse model is more severe. This difference in severity was consistently found when examining the data from OCT, ERGs and VGSA, and again mirrors observations in patients with BBS10 versus BBS1 (Grudzinska Pechhacker et al., 2021). The fidelity of the phenotype with respect to endpoints similar to those used in humans makes this model ideal for testing the ability of preclinical treatments to slow or reverse retinal degeneration in BBS10.

## RESULTS

### Deletion of the mouse *Bbs10* gene

*Bbs10^−/−^* mice were obtained from The Jackson Laboratory. These mice had been developed using an embryonic stem cell line from the Knockout Mouse Project (KOMP) repository [KOMP embryonic stem cell line *Bbs10^tm1(KOMP)Vlcg^* #052680-UCD]. The mice were acquired from the KOMP2 Center at The Jackson Laboratory [now distributed through the Mutant Mouse Resource and Research Center (MMRRC)] [C57BL/6N-Bbs10tm1.1(KOMP)Vlcg/JMmucd #046771-UCD]. Mouse *Bbs10* is composed of two exons, and, in this model, most of exon 1 and most of exon 2 are deleted, eliminating most of the protein-coding region of the *Bbs10* gene. As a result, *Bbs10^−/−^* mice do not produce any *Bbs10* mRNA (Fig. S1). *Bbs10* transcripts were detected in whole eyes of 1-month-old *Bbs10^+/−^* mice, but not in those of *Bbs10^−/−^* littermates (Fig. S1). *Bbs10^−/−^* males are sterile but females are fertile, as is the case for other BBS mouse models (Cognard et al., 2015; Datta et al., 2015; Davis et al., 2007; Nishimura et al., 2004; Zhang et al., 2011, 2013). Therefore, all mice must be produced using *Bbs10^+/−^*×*Bbs10^+/−^* or *Bbs10^+/−^*×*Bbs10^−/−^* crosses, with all males being heterozygous and females being either heterozygous or homozygous mutant. All mice were genotyped using DNA obtained from tail snips. The forward primer for detecting the wild-type (WT) allele is complementary to a region of exon 2 that is deleted in knockout mice, whereas the forward primer for detecting the knockout allele is complementary to the sequence in the cassette inserted for generating the deletion. The reverse primer is complementary to a sequence near the end of exon 2, and the expected PCR products were 445 bp and 262 bp, respectively. *Bbs10*^−/−^ pups are those that display a single 262 bp PCR product, WT pups are those that display a single 445 bp PCR product, and heterozygous pups are those that display both bands (Fig. S2). A very small band that appears in all samples at <100 bp likely represents primer dimers. Given that only homozygous knockout mice display a retinal phenotype, both *Bbs10^+/+^* and *Bbs10^+/−^* littermates were used as controls.

### *Bbs10^−/−^* mice are smaller at birth and become progressively larger than controls

**Fig. 1.**
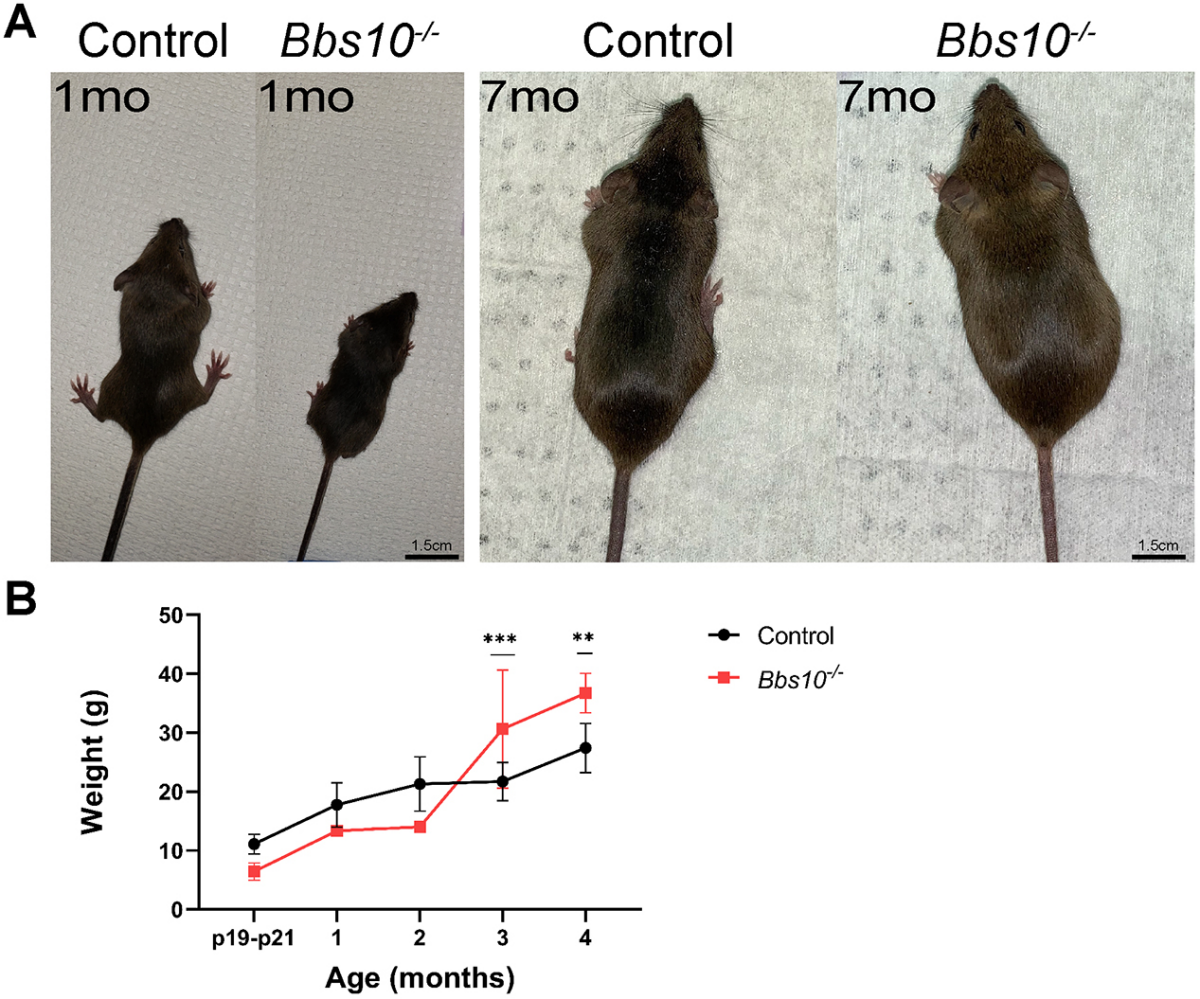
**In *Bbs10^−/−^* mice, weight is altered relative to that in controls.** (A) Overall sizes of representative *Bbs10^−/−^* and control [wild-type (WT)] mice at 1 and 7 months of age. Note the slightly narrower abdominal area of the control mouse at 7 months of age. (B) Quantification of the weights of the two groups from P19 to 4 months of age. Significance of differences in means of control and *Bbs10^−/−^* mice was determined for each age using two-way ANOVA with Šídák's multiple comparisons test. **P<0.01, ***P<0.001.

At 1 month of age, *Bbs10^−/−^* mice were visibly smaller than WT and heterozygous *Bbs10^+/−^* littermates (Fig. 1A). At P19-P21, control pups averaged 11.11 g (±1.68 g s.d.) (*n*=11), whereas *Bbs10^−/−^* pups averaged only 6.44 g (±1.45 g s.d.) (*n*=7) (Fig. 1B). By 4 months of age, the average weight of the control mice was 27.43 g (±4.20 g s.d.) (*n*=12), whereas the average weight of the *Bbs10^+/−^* mice was 36.73 g (±3.35 g s.d.) (*n*=3) (Fig. 1B), and this difference persisted throughout life. Statistical comparisons between the two mouse groups from P19 to 4 months of age using a two-way ANOVA with Šídák's multiple comparisons test showed that the weights differ significantly at ages 3 (*P*<0.001) and 4 (*P*<0.01) months, but not at P19-P21. The latter result could potentially be due to small sample size. Mortality rates for the *Bbs10^−/−^* pups were high, and thus special husbandry was needed. This included feeding nursing mothers and their pups DietGel and delaying weaning to as late as P35. Progressive weight abnormalities are similar to those observed in patients with BBS10.

### In *Bbs10^−/−^* mice, the outer nuclear layer of the retina progressively becomes thinner

**Fig. 2.**
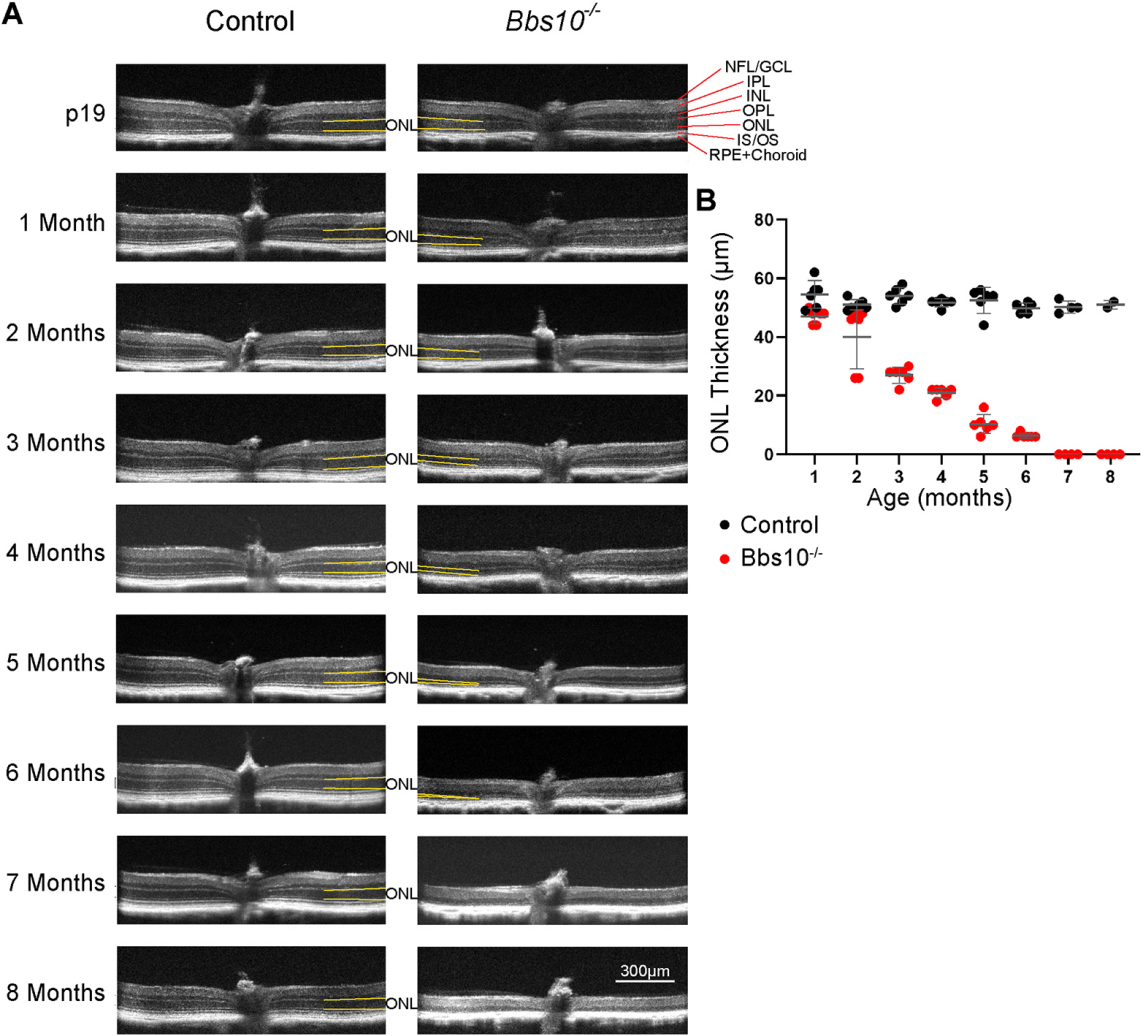
**In *Bbs10^−/−^* mice, the outer nuclear layer (ONL) of the retina progressively becomes thinner than that of controls.** (A) Optical coherence tomography (OCT) images of control and *Bbs10^−/−^* mice from P19 to 8 months. (B) Quantification of ONL thickness in control mice and *Bbs10^−/−^* mice at various ages. Significance was determined for each group by a two-way ANOVA with Šídák's multiple comparisons test of the means. **P<0.01, ****P<0.0001.

OCT is a noninvasive imaging technique that can be used to observe the layers of the retina *in vivo* (Fischer et al., 2009). The thickness of the outer nuclear layer (ONL) is a common indicator of retinal degeneration, as it contains the nuclei of rod and cone photoreceptors (Lujan et al., 2015). Thinning of the ONL indicates that the number of photoreceptors is reduced and is associated with retinal degeneration (Fig. 2A, brackets). Whereas at 1 month of age, the control mice had an average ONL thickness of 55.0 µm (±2.0 µm s.e.m.), in the *Bbs10^−/−^* mice it was 47.0 µm (±1.0 µm s.e.m.), or 85% of that in the controls. In the control mice, the thickness of the ONL remained between 50.0 µm and 55.0 µm from 1 month to 8 months of age. In contrast, in the *Bbs10^−/−^* mice, the ONL thinned progressively during this time. In mice at 3 months of age, the average thickness of the *Bbs10^−/−^* mouse ONL was 28.0 µm (±1.0 µm s.e.m.) versus 54.0 µm (±1.0 µm) in control mice, or 50% of the thickness in control mice. By 7 months of age, *Bbs10^−/−^* mice had no distinguishable ONL (Fig. 2B). In fact, histological analysis showed that by 7 months of age *Bbs10^−/−^* mice retained only a single layer of cells (Fig. S3). Thus, OCT demonstrated that *Bbs10^−/−^* mice undergo a significant loss of ONL during their lifetime.

### In young *Bbs10^−/−^* mice, the retina is disorganized and markers of photoreceptors are absent

**Fig. 3.**
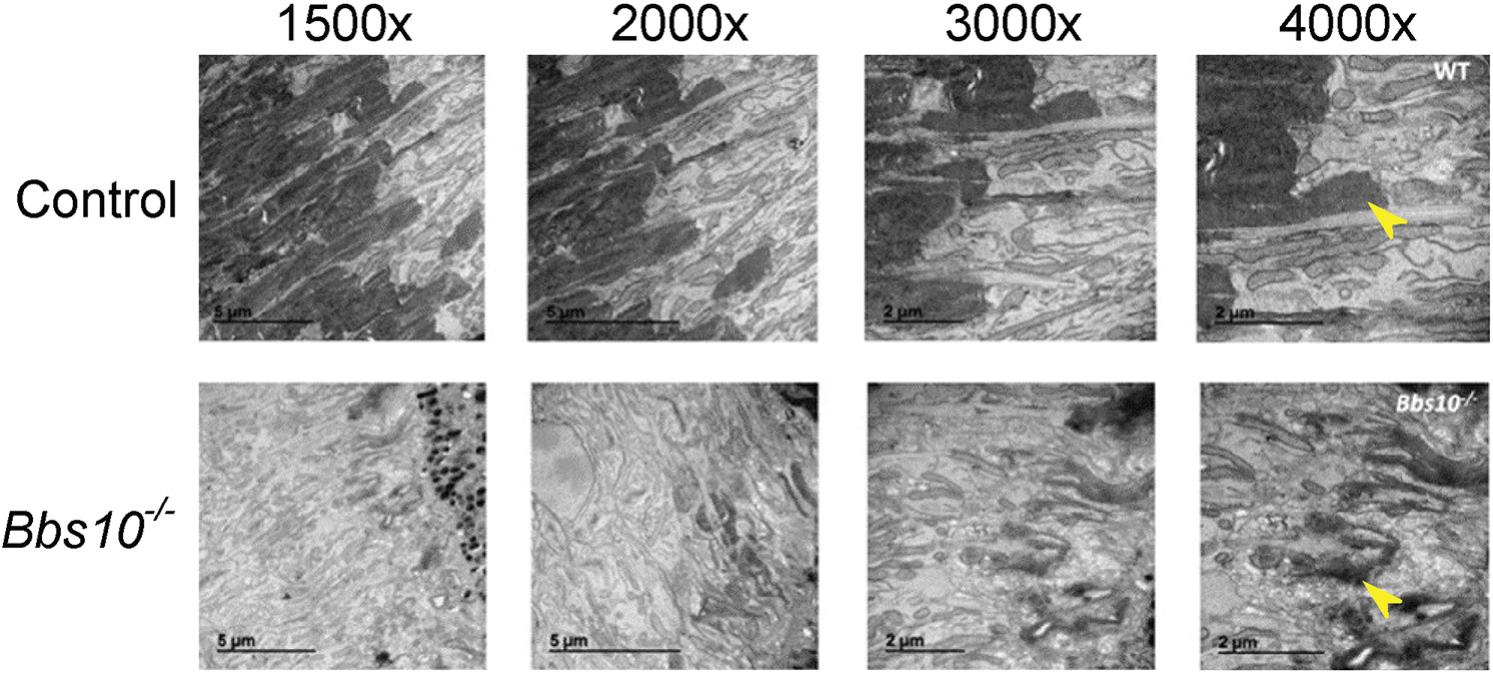
**In *Bbs10^−/−^* mice, photoreceptor outer segments are disorganized at P15.** Comparison of transmission electron micrographs of retinas from a control and a *Bbs10^−/−^* mouse at the indicated magnifications. Arrowheads at 4000× point to outer segment discs in the two genotypes. Note the organization of the large outer segment discs of the control mouse (top row, arrowhead) and the relative disorganization of their counterparts in the *Bbs10^−/−^* mouse.

In control mice, the outer segment of the photoreceptors is highly organized, with clearly defined horizontally organized discs that are visible by transmission electron microscopy (TEM) (Fig. 3, top row, arrowhead). These structures are abnormal in *Bbs10^−/−^* mice as early as P15. In addition to lacking this disc organization, the outer segments lack any remnant of polarity. This early anatomic abnormality has implications for the role of BBS10 in early formation and maintenance of the retinal photoreceptors' outer segments and makes it clear that early treatment of BBS10 in humans may be optimal.

**Fig. 4.**
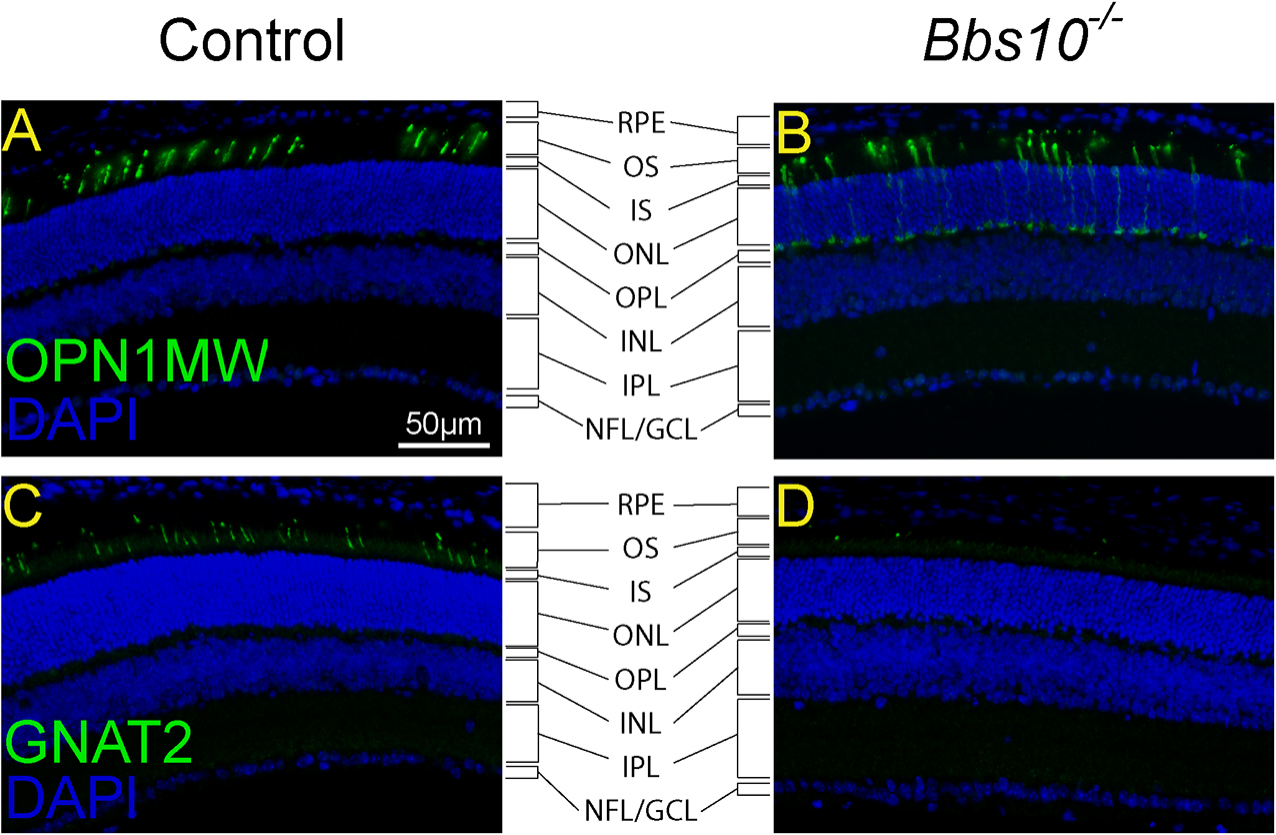
**In *Bbs10^−/−^* mice, localization of cone proteins is disorganized compared to that in controls.** (A-D) OPN1MW (A,B) and GNAT2 (C,D) are cone-specific proteins that are normally present in WT outer segments. The images are counterstained with 4′,6-diamidino-2-phenylindole (DAPI). It should be noted when analyzing localization that the proximal end of cone OS is slightly ‘embedded’ in the (anatomical) IS. The distal end of the cone OS typically ends ‘sooner’ than that of the rod OS. GCL, ganglion cell later; INL, inner nuclear layer; IPL, inner plexiform layer; IS, inner segment; NFL, nerve fiber layer; ONL, outer nuclear layer; OPL, outer plexiform layer; OS, outer segment; RPE, retinal pigmented epithelium.

The above findings are consistent with immunofluorescence images of the retinas of P21 mice, which revealed that some cone markers are mislocalized and others are absent. OPN1MW, the protein pigment for medium-wave-sensitive opsin 1, is a cone photoreceptor pigment (Deeb, 2004). Although OPN1MW normally localized to the outer segment in healthy cone photoreceptors (Fig. 4A), as previously reported (Datta et al., 2015), in young P21 *Bbs10^−/−^* mice, it was dispersed throughout the cone photoreceptor cells, including in the inner segments and at the junction of the ONL and the outer plexiform layer (OPL) in the synaptic terminal (Fig. 4B). In contrast, the cone-specific protein, GNAT2, the alpha subunit of cone transducin (Aligianis, 2002), was nearly absent from *Bbs10^−/−^* mouse photoreceptor layers by P21 (Fig. 4C,D). In previous studies of mouse BBS models (Datta et al., 2015), GNAT2 was found to be mislocalized to the inner segment, as was the case here with OPN1MW. A lack of properly localized cone-specific proteins at such an early age indicates that onset of the BBS10 disease phenotype is very early in life.

### In young *Bbs10^−/−^* mice, ERG amplitudes are low and the 5 Hz flicker response is absent

**Fig. 5.**
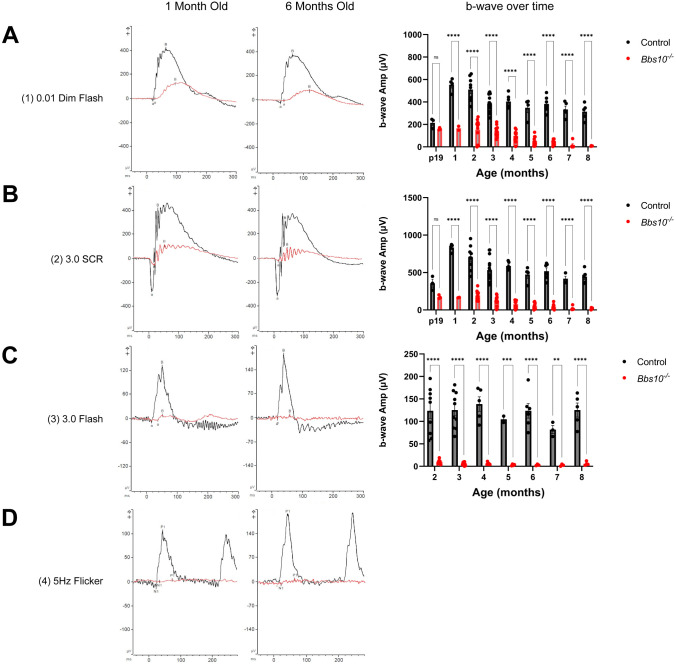
**Electroretinograms (ERGs) of *Bbs10^−/−^* mice are abnormal.** (A-D) Shown for each are overlays of ERG waveforms for a representative control and *Bbs10^−/−^* mouse after the indicated form of stimulation, in mice at 1 month of age (left column) and 6 months of age (middle column), and amplitudes of the b-wave over time for that condition (right column). (A) Under the 0.01 cd•s/m^2^ dark-adapted dim-flash condition. (B) Under the 3.0 cd•s/m^2^ dark-adapted condition to elicit that standard combined response (SCR). (C) Under the 3.0 cd•s/m^2^ light-adapted bright-flash condition. (D) Under the 5 Hz flicker light-adapted condition. The waveforms were not quantified due to the lack of signal beyond background in *Bbs10^−/−^* mice. Significance was assessed using a two-way ANOVA with Šídák's multiple comparisons test to compare the means of each genotype at each age. **P<0.01, ***P<0.001, ****P<0.0001; ns, not significant.

ERGs are used to measure the collective electrical output of all photoreceptors of the retina (Robson et al., 2018), following stimulation with a series of light flashes of varying intensities and under different lighting conditions. The differences in lighting conditions make it possible to elicit separate responses from rods and cones. When the eye is dark adapted, rods are the predominant photoreceptor that fires in response to a dim-light stimulus (Robson et al., 2018); when it is light adapted, cones are the predominant photoreceptors that respond. Using a modified International Society for Clinical Electrophysiology of Vision (ISCEV) standard protocol (Mcculloch et al., 2015), we tested four conditions. (1) Dark-adapted retinas were stimulated with 0.01 cd•s/m^2^ (dim) flashes (Fig. 5A), to which only rods respond. (2) Dark-adapted retinas were stimulated with 3.0 cd•s/m^2^ (bright) flashes (Fig. 5B), which elicit responses from both rods and cones (Robson et al., 2018). The 3.0 cd•s/m^2^ bright flash elicits the standard combined response (SCR), which represents a combination of responses from rod and cones. Of all conditions tested, this stimulation of dark-adapted retinas with a bright flash caused the largest number of photoreceptors to fire and thus elicited the largest response (Fig. 5B). (3) Light-adapted retinas were stimulated with 3.0 cd•s/m^2^ (bright) single flashes (Fig. 5C), which elicits response primarily from cones. (4) Light-adapted retinas were stimulated with a 5 Hz flicker (Fig. 5D). The 5 Hz flicker stimulus uses the same 3.0 cd•s/m^2^ flash, but at a higher frequency (5 Hz). This light-adapted condition has been shown to elicit a predominately cone response in mice (Seeliger et al., 2001; Tanimoto et al., 2015). For each condition, both ERG a-waves and b-waves were analyzed; the a-waves represent primarily the firing of photoreceptors, whereas the b-waves represent primarily the firing of bipolar cells after synapse with the photoreceptors (Robson et al., 2018). Given that photoreceptors form synapses with bipolar cells and transmit visual signals to them, the b-wave follows the a-wave (Robson et al., 2018).

Compared to control mice, the *Bbs10^−/−^* mice showed reduced SCR ERGs. Specifically, in *Bbs10^−/−^* mice at 1 month of age, the b-wave amplitudes were 30.1% of those in control mice for the response to the 0.01 dim flash and 19.9% of those for control mice for the response to the 3.0 bright flash (Fig. 5A,B). The *Bbs10^−/−^* mice lacked a light-adapted 5 Hz flicker response at this age; electrical activity of the retina was indistinguishable from background noise (Fig. 5D). Of note, whereas the *Bbs10^−/−^* mice lacked a robust ERG cone response at a young age (Fig. 5C,D), histological analysis showed that cones were anatomically present at P21 (Fig. 4). In *Bbs10^−/−^* mice, the amplitude of b-waves triggered in the light-adapted mice in response to the 3.0 bright flash was significantly reduced; it was only 0.083% of that in control mice at 2 months of age. This barely recordable light-adapted 3.0 bright-flash b-wave amplitude became nonrecordable at 5 months of age (Fig. 5C). The light-adapted 5 Hz flicker was not plotted due to the nonrecordable nature at the earliest ages tested (Fig. 5D). The ERG data indicate that the *Bbs10^−/−^* mice had severe photoreceptor dysfunction at an early age and that these worsened as the mice aged.

### In *Bbs10^−/−^* mice, functional vision is decreased compared to that of controls

An ERG tests whether photoreceptors are functional but not whether vision is. It is possible to have useful functional vision in the absence of a recordable ERG because the mass response detected by the ERG is insensitive to small numbers of functional photoreceptors, and just a few light-sensitive photoreceptors can confer a small field of very useful vision. We developed a VGSA to test functional vision of the *Bbs10^−/−^* mice and compare it to that in control mice. A highly visible platform was placed in random locations in a pool of water, and mice needed to locate the platform via vision and swim to it. Swim time was measured as time to platform (TTP), and this value was shorter for mice with normal vision than for mice with retinal degeneration and reduced vision.

Analysis was performed on five affected *Bbs10^−/−^* mice and eight control mice. The average TTP for control mice aged 6 months was 5.07 s (±0.67 s s.e.m.) when light adapted and 5.50 s (±0.63 s s.e.m.) when dark adapted. At the age of 9 months, the average TTP for control mice was 3.33 s (±0.39 s s.e.m.) in the light and 4.34 s (±0.82 s s.e.m.) in the dark. These averages were not significantly different, showing that functional vision remained stable in the control mice as they aged. These findings also show that the VGSA provided a reproducible measure of visual function in mice.

**Fig. 6.**
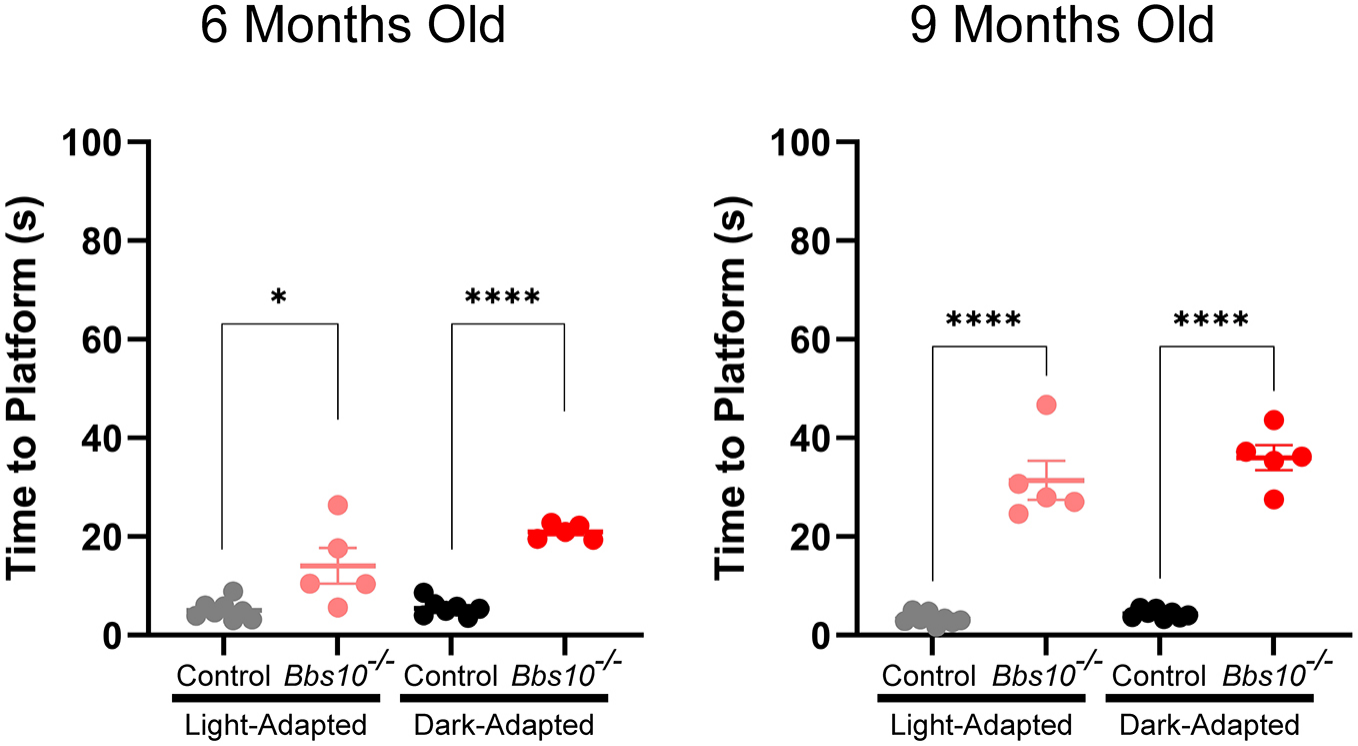
***Bbs10^−/−^* mice have reduced functional vision compared to control mice.** Quantification of time taken for control and *Bbs10^−/−^* mice to complete the visually guided swim assay (VGSA) under light-adapted and dark-adapted conditions, at 6 (left) and 9 (right) months of age. Significance was assessed using unpaired two-tailed Student's *t*-tests to compare mice of the two genotypes at these ages and under these light conditions. *P<0.05, ****P<0.0001.

In the case of the 6-month-old *Bbs10^−/−^* mice, the average TTP for light-adapted mice was 14.08 s (±3.62 s s.e.m.) and for dark-adapted mice it was 20.97 s (±0.68 s s.e.m.) (Fig. 6). In both conditions, the average TTP was significantly different from that in controls, with light-adapted *Bbs10^−/−^* mice taking 2.6 times longer, and dark-adapted mice taking 4.1 times longer, to reach the platform than the controls (*P*=0.0102 and *P*≤0.0001 for light-adapted and dark-adapted mice, respectively). There was also a trend toward a difference (*P*=0.0982) between light and dark conditions for the *Bbs10^−/−^* mice at this age. Although not significant, this trend suggests that *Bbs10^−/−^* mice have worse vision in the dark than in the light at this young age. By the age of 9 months, the *Bbs10^−/−^* mice averaged a TTP of 31.40 s (±3.94 s s.e.m.) when light adapted and 35.99 s (±2.57 s s.e.m.) when dark adapted, times that are significantly longer than those for controls of the same age tested in the same lighting conditions (3.33 s and 4.34 s, respectively). Again, the difference between the control and *Bbs10^−/−^* mice was stark, with *Bbs10^−/−^* mice taking 9.4 times longer than controls when light adapted and 8.3 times longer when dark adapted (*P*≤0.0001).

The *Bbs10^−/−^* mice also displayed progressive vision loss. The difference in TTP between young and old *Bbs10^−/−^* mice was significant for the light-adapted and dark-adapted conditions (*P*=0.0119 and *P*=0.0005, respectively). The times for 9-month-old light-adapted *Bbs10^−/−^* mice were 2.2 times as long as those for 6-month-old mice. Times for dark-adapted 9-month-old *Bbs10*^−/−^ mice were 1.7 times as long as those for 6-month-old mice. The VGSA is able to show a further progression in vision loss of these mice where an ERG cannot. When *Bbs10^−/−^* mice were 6 months of age, the light-adapted ERG was indistinguishable from background noise. However, the VGSA revealed a significant progression of retinal degeneration from 6 to 9 months of age. Reasons for this could be differences in the sensitivities of the VGSA and ERG, with a floor effect in the ERG. Here, the floor effect refers to the lowest values a technique or device can record. Below the floor of the test, there may still be data and significant change, but it cannot be measured by the techniques employed.

### In *Bbs10^−/−^* mice, retinal degeneration is more severe than that in the *Bbs1^M390R/M390R^* BBS1 mouse model

**Fig. 7.**
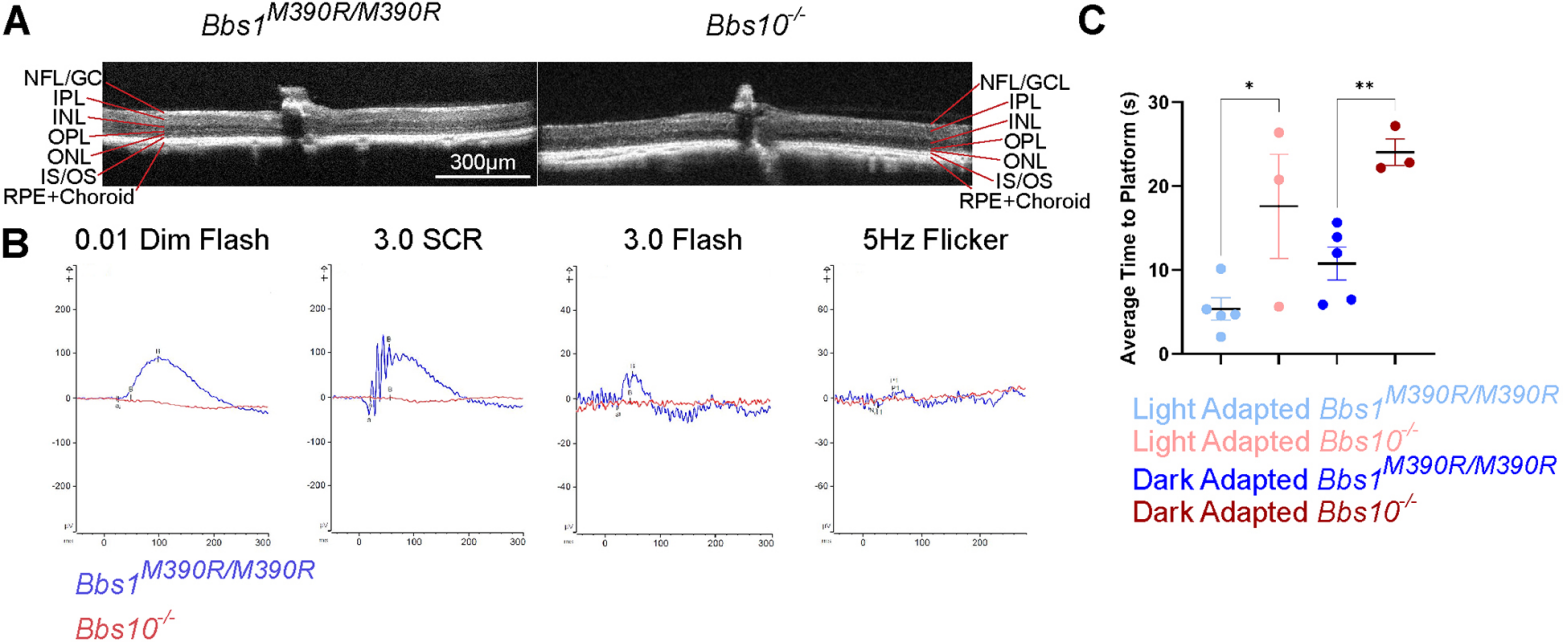
**The retinal degeneration in *Bbs10^−/−^* mice is more severe than that in *Bbs1^M390R/M390R^* mice at 6** **months of age.** (A) OCT images of 6-month-old mice of the two genotypes. (B) Overlays of ERG waveform under the indicated conditions, for *Bbs1^M390R/M39R^* and *Bbs10^−/−^* mice. (C) Outcomes of VGSA for *Bbs1^M390R/M390R^* and *Bbs10^−/−^* mice. Significance was assessed using unpaired two-tailed Student's *t*-tests, comparing the two genotypes under different conditions. *P<0.05, **P<0.01.

In humans, BBS1 and BBS10 account for ∼50% of all BBS cases, but the progression of BBS1 is slower than that of BBS10 (Grudzinska Pechhacker et al., 2021). Our group had previously developed a mouse model of the most common BBS1-causing mutation, homozygous M390R (Davis et al., 2007), and it was available for comparison to the BBS10 mouse model described here. For this comparison, both mouse models were crossed onto the 129/SvJ background for at least seven generations. At 6 months of age, the *Bbs10^−/−^* mice were compared to *Bbs1^M390R/M390R^* mice. OCT revealed that whereas the *Bbs10^−/−^* mice completely lacked the ONL at 6 months of age, the *Bbs1^M390R/M390R^* mice retained a thin layer (Fig. 7A). ERGs taken from the retinas of 6-month-old *Bbs1^M390R/M390R^* mice showed that the amplitudes were distinctly higher than those from the retinas of same-aged *Bbs10^−/−^* mice. Of note, *Bbs1^M390R/M390R^* mice had a 5 Hz flicker response that was detectable at 6 months, whereas the *Bbs10^−/−^* mice lacked this response even at P30 (Fig. 5D, Fig. 7B). At 6 months of age, functional vision of *Bbs1^M390R/M390R^* mice was also better than that of age-matched *Bbs10^−/−^* mice (Fig. 7C). Results from light-adapted and dark-adapted conditions demonstrate that the mouse models recapitulate the human disease, with BBS10 being more severe than BBS1.

## DISCUSSION

This study sought to characterize the retinal phenotype in a new mouse model of BBS10, one of the most common types of BBS in humans (Grudzinska Pechhacker et al., 2021; Stoetzel et al., 2006). The *Bbs10^−/−^* mice displayed increased weight gain, male infertility and severe retinal degeneration, similar to other genetic types of BBS mouse models (Cognard et al., 2015; Datta et al., 2015; Davis et al., 2007; Hsu et al., 2017; Zhang et al., 2011, 2013). In addition, they showed a progressive retinal degeneration that is characteristic of BBS and is anatomically and functionally distinguishable from the phenotypes of the BBS1 mouse model (Davis et al., 2007).

From at least P21, *Bbs10^−/−^* mice had mislocalized cone-specific proteins (OPN1MW and GNAT2). The light-adapted response to a 5 Hz flicker stimulus, which is predominately a cone response, was completely absent in *Bbs10^−/−^* mice at the earliest age tested (P30). At this early age, however, the rod response was present. This suggests a cone-rod dystrophy, which is unusual for RP and many types of BBS (Iannaccone et al., 1996; Weihbrecht et al., 2017). The lack of response in the cone ERG could result from loss of functional cone transducin, a protein needed for transmitting signals from cone photoreceptors (Aligianis, 2002), or a reduction in the amplitude of the electrical signal to below the threshold of detection on ERG. The latter could be due to a reduction in the number of cones that fire or a low amplitude of the electrical impulses from individual cones. Our discovery that the outer segments were disordered and lacked GNAT2 protein at early ages suggests that BBS10 protein is required early in photoreceptor formation as well as in photoreceptor maintenance. Levels of BBS proteins start to increase at ∼P4-P6, peak at P15 when the outer segment begins to mature, and decrease to maintenance levels after P15 (Hsu et al., 2017). The abnormal anatomical phenotype in the *Bbs10^−/−^* mice suggests that BBS10 is also important during this early development period.

We speculate that the cause of the mislocalization of OPN1MW and the complete lack of GNAT2 is malfunction of the BBSome. Immunohistochemistry in a mouse model of BBS type 17 (BBS17) revealed that these proteins are mislocalized from the outer to the inner segment (Datta et al., 2015). Proteomics data for the same mouse are consistent with this finding, revealing a clear lack of cone-specific markers in the outer segment (Datta et al., 2015). BBS17 facilitates shuttling of the BBSome out of the cilia (Seo et al., 2011). Moreover, data from a mouse model of BBS type 5 (BBS5), the *Bbs5^−/−^* mouse, show that both OPN1MW and GNAT2, as well as other cone-specific protein markers, are mislocalized to the inner segment (Bales et al., 2020). BBS5 is a part of the BBSome. Additionally, ERGs showed that these mice have reduced cone function and that the outer segments of the photoreceptors are disorganized, features very similar to those reported here for the *Bbs10^−/−^* mouse. Along with other proteins of the BBSome chaperonin-like complex, BBS10 is responsible for assembling the full BBSome, which aids in ciliary and cellular trafficking (Hsu et al., 2017; Seo et al., 2010, 2011). A well-known example of faulty trafficking in the context of BBSome dysfunction is a failure of the leptin receptor to reach the cellular membrane. The resulting loss of leptin signaling typically leads to obesity. Indeed, the defect in the trafficking of this receptor is a common feature in genetic forms of obesity, including in patients with BBS (Rahmouni et al., 2008; Seo et al., 2009). It is possible that, at P15, OPN1MW requires the functional BBSome for transport to the outer segment. GNAT2 is a protein that forms a complex with two other subunits to produce a full functional protein, cone transducin (Aligianis, 2002). It too seems to be mislocalized in the absence of a functional BBSome (as in the case of BBS5), or when the BBSome is not fully functional (as in the case of BBS17), and thus it may be mislocalized because of the loss of BBS10, which is required for BBSome assembly (Seo et al., 2010). Our results show that GNAT2 is almost entirely absent from retinas of the *Bbs10^−/−^* mice, and this could result from protein degradation. Without BBS10 to direct the formation of a functional BBSome, either cone transducin cannot form or is mislocalized or degraded during these early stages of eye development, and it is therefore only detected at very low levels by immunohistochemistry in photoreceptors at P21.

One interesting result was the better VGSA swim time in light-adapted versus dark-adapted *Bbs10^−/−^* mice. At 6 months of age, *Bbs10^−/−^* mice performed the VGSA faster in bright light than in dark light, having an average TTP much closer to that of control mice in the light than in the dark. This indicates that these mice retain functional vision in bright light early in life, despite the lack of GNAT2 in cones and lack of a light-adapted cone-predominant 5 Hz response on ERG. Because the ERG represents a mass retinal response and the mouse retina contains far more rods than cones (Fu and Yau, 2007), a reduction in the number of rods may result in a diminished ERG amplitude, yet the effect of a proportional reduction in the number of cones on electrical output may be an ERG amplitude too small to be detected by current technologies. It is possible that a small cohort of functioning cones were not detectable on ERG because the sum of their amplitudes was not sufficiently robust for detection. However, this small cohort of functional cones in young mice could be adequate for navigation in the swim assay. By 6 months, in the dark-adapted swim condition, which tests predominately rods, the dark-adapted ERG is of very low amplitude but still recordable.

Although the persistent barely recordable dark-adapted ERG amplitudes suggest that the *Bbs10^−/−^* mice should have a better dark-adapted TTP compared to light-adapted TTP where light-adapted ERG remains nonrecordable, the rods that respond on ERG may not fully translate to functional vision. This could be due to a problem later in visual processing that cannot be picked up by ERG, or it could be a result of a small foci of rods in the periphery of the retina numerous enough to achieve a recordable ERG waveform but not positioned well enough to be used for navigation. In the light-adapted condition, bright room lights are used, sufficient for a wide range of cone sensitivities. In the dark-adapted condition, only a dim-red illumination is present in the room, which might not be sufficient for rod vision, while the flashes of white light used for ERG can cause recordable stimulation. Overall, the seemingly paradoxical VGSA results could be explained by very small populations of functional cones improving navigation in bright light, with small regions of rods that respond to stimulation on ERG of limited navigational value in very dark surroundings. More data are needed to draw a definitive conclusion.

Although mutations in BBS disease genes generally cause syndromic forms of retinal degeneration, isolated RP has been reported in association with mutations in *BBS1* (Estrada-Cuzcano et al., 2012 a,b) and *BBS10* (Grudzinska Pechhacker et al., 2021). The retinal phenotype of BBS1 is typically described as RP-like rod-cone dystrophy (Grudzinska Pechhacker et al., 2021); however, BBS10 may occur as a cone-rod dystrophy, or even an isolated cone dystrophy (Grudzinska Pechhacker et al., 2021). Patients with BBS10 often present with simultaneous night blindness and peripheral field constriction, as well as reduced central vision, a devastating combination (Grudzinska Pechhacker et al., 2021). Previous studies on BBS in humans indicate that these diseases usually present as a rod-cone dystrophy, and thus the disease pattern in BBS10 is fairly unique (Grudzinska Pechhacker et al., 2021; Iannaccone et al., 1996; Weihbrecht et al., 2017). However, recent evidence suggests that other forms of BBS may also present as a cone-rod dystrophy. One example is a case of two brothers who have been diagnosed with BBS7, and both display a cone-rod dystrophy (Aleman et al., 2021). Additionally, a mouse knockout model of BBS5 developed a cone-rod dystrophy as well as displayed mislocalization of cone-specific proteins such as OPN1SW, OPN1MW and GNAT2 (Bales et al., 2020); we report that OPN1MW and GNAT2 are mislocalized in *Bbs10^−/−^* mice. It would seem that, in the models of BBS10 and BBS5, both of these proteins are mislocalized to the inner, rather than the outer, photoreceptor segment. This implies that the disease-causing mechanisms are similar in BBS5, BBS7 and BBS10, and that they lead to a lack of trafficking of the proteins that are necessary for proper cone function. Although BBS10 is not itself a component of the BBSome, it is required for the sequential assembly of the BBSome, and both are located inside the cilia in the outer segment of the photoreceptor (Hsu et al., 2021; Zhang et al., 2012). A relationship between BBS5, BBS7 and BBS10 should be explored, as should their role in mediating the transport of necessary cone proteins into the outer segment for proper photoreceptor maintenance.

The phenotype of *Bbs10^−/−^* mice is similar to that of humans, making them an excellent model for use in preclinical trials of potential therapeutics. It is worth noting that the mouse models used in this study are not both knockouts. The BBS10 mouse model is a full knockout that produces no *Bbs10* mRNA; in contrast, the BBS1 mouse harbors a substitution mutation that changes just one amino acid in the protein. This may contribute to the difference in severity of the phenotypes of the models. However, this difference is an accurate comparison of the two diseases for humans; whereas the most common cause of BBS10 in humans is a single-nucleotide insertion that results in premature termination and the loss of protein expression (Stoetzel et al., 2006), the most common cause of BBS1 is the same amino acid substitution mutation reported in the mouse model here (Davis et al., 2007; Mykytyn et al., 2002). This substitution mutation may leave some part of a functioning protein, albeit abnormal, whereas the BBS10 mutation produces no protein whatsoever. Thus, a partially functional *BBS1* gene may result in a partially functional BBSome, whereas a completely nonfunctional *BBS10* gene may lead to little or no BBSome formation, explaining the more severe BBS10 retinal phenotype in mouse and man.

In conclusion, a knockout mouse model of BBS10 recapitulates the retinal degeneration in human patients and shares features of that condition. Comparison to a model of BBS1 demonstrates that different subtypes of BBS will likely require different rescue strategies and timing of treatment. In the retinas of *Bbs10^−/−^* mice, the rods and cones are present early in life but are abnormal and degenerate over time. The early nonrecordable light-adapted 5 Hz flicker ERG offers a robust endpoint for therapeutic rescue studies. We are currently exploring disease and treatment timelines to help clinicians and patients understand when treatment will be most effective. The fact that useful functional vision in the light is possible in mice even when ERGs are nonrecordable suggests that the rescue of even a small percentage of cones could prove beneficial in patients.

## MATERIALS AND METHODS

### Animals

Experiments were approved by the Institutional Animal Care and Use Committee (IACUC) at the University of Iowa and conducted following the recommendations in the Guide for the Care and Use of Laboratory Animals of the National Institutes of Health. Mice were maintained on a standard 12/12 h light/dark cycle with food and water provided *ad libitum*. DietGel (ClearH2O, Westbrook, ME, USA) was frequently fed to pups as *Bbs10*^−/−^ mice are typically runts and required special husbandry. The breeders from which the line was started contained the *Rd8* mutation in *Crb1*; this was bred out early in the process. Genetic testing as well as genotyping for the *Bbs10* allele confirmed that the *Crb1* gene was WT. WT 129/SvJ mice were obtained from The Jackson Laboratory (Bar Harbor, ME, USA).

### OCT

OCT was performed using a Bioptigen OCT system with a small rodent lens (Research Triangle Park, NC, USA), as previously described (Drack et al., 2012). Mice were anesthetized with a mixture of ketamine (87.5 mg/kg) and xylazine (2.5 mg/kg), and pupils were dilated with tropicamide 1%. The OCT images are aligned horizontally and represent a slice in the frontal plane of the mouse. All images are a central OCT scan from temporal to nasal sides of the mouse. For all images, measurements of the ONL were taken from 300 µm on either side of the optic nerve, using the in-software calipers provided by Bioptigen.

### Microscopic anatomy

Mice were sacrificed at the indicated times. Retinal sections were incubated with rabbit anti-OPN1MW polyclonal antibody (EMD Millipore, AB5405; 1:200) and rabbit anti-GNAT2 polyclonal antibody (Abcam, ab97501; 1:100). Immunofluorescence was performed as described previously (Datta et al., 2021, 2019, 2020). TEM was performed as described previously (Hsu et al., 2017).

### ERGs

Full-field ERGs were conducted within 2 weeks of each swim assay, using a Celeris Diagnosys system (Diagnosys LLC, Lowell, MA, USA). The mice were dark adapted overnight before ERG was conducted. Mice were anesthetized with a mixture of ketamine (87.5 mg/kg) and xylazine (2.5 mg/kg). The mice received 0.1 ml of the mixture per 20 g body weight. ERGs were recorded from the corneal surface of each eye simultaneously, after the pupils were dilated with 1% tropicamide, using Diagnosys Celeris touch stimulator electrodes. Gonak gel (Akorn, Lake Forest, IL, USA) was placed on the cornea of each eye before the electrode was positioned. Light flashes were produced by the touch stimulator electrodes. Dim-red light was used to illuminate the room until dark-adapted testing was completed. A modified ISCEV protocol was used. A dim flash of 0.01 cd•s/m^2^ was first used to stimulate rods, followed by a bright flash at 3.0 cd•s/m^2^ to measure the SCR of the rods and cones. Mice were then light adapted for 10 min, after which they were tested with a bright flash at 3.0 cd•s/m^2^ followed by a flicker of 5 Hz. For most measurements, 15 sweeps were taken per condition; for the 5 Hz flicker, 20 sweeps were taken. ERG results were analyzed following data collection, using the Diagnosys software to eliminate sweeps that show interference, such as mouse movements or ambient electric signal. Any test in which more than five sweeps were needed to eliminate interference was considered null, and it was repeated for cleaner results. The light-adapted protocol tests cones, especially the 5 Hz flicker, which in mice elicits a response from only cones (Tanimoto et al., 2015).

### VGSA

A modified Morris water maze (Morris, 1984) incorporating some features of the mouse swim assay developed by Pang et al. (Pang et al., 2008) was utilized to test functional vision. A plastic swimming pool was used for swimming. The pool diameter measures 35 inches at the bottom, 38 inches at the 4 inch water level, and 39 inches at the top. A heavy 3 inch-diameter PVC tube with rubber caps on both ends was used as a platform for the mice to climb onto to end the trial. The platform was the same on both ends, so it could be flipped between trials to mitigate any odor that might aid in platform localization. A ‘flag’, consisting of a small paper American flag taped to a wooden stick, was attached to the side of the platform to make it more visible to the mice. Platform location consisted of eight equally spaced pre-set locations. The order of the locations of the platform was determined prior to the day's experiment and was randomly determined each day.

The swim protocol consisted of 4 light-adapted training days, followed by 4 light-adapted testing days, then 2 days of dark-adapted training and finally 4 days of dark-adapted testing. Light adaptation was performed in a brightly lit room of typical fluorescent ceiling lights (292 lux). Dark adaptation was performed in the same room, with the lights off and dim-red lights to enable the observers to watch the experiments (0.013 lux). An infrared lamp was aimed at the pool, and the location of the mice was determined using night-vision goggles.

During each testing day of swimming, mice performed five swim trials to five different randomly selected platform locations. These platforms were the same for all mice in the test group. A limit of 60 s was set for swim TTPs to prevent mice from becoming fatigued; if the platform was not attained by 60 s, the mouse was gently removed from the pool, and the TTP for that particular trial was recorded as 60 s.

### Genotyping

Genotyping was performed using mouse tail snips collected at P14. DNA was extracted, and *Bbs10* was amplified using WT and knockout forward primers and the same reverse primer (Table S1).

### RT-PCR

RNA was extracted from whole-eye lysates, and it was amplified using the SuperScript VILO Master Mix to create a cDNA library. After this, PCR was run on the cDNA amplified by the indicated primers (Table S1). Actin was used as a control.

### Statistical analysis

Statistical analysis and figure creation were performed using GraphPad Prism and Adobe Illustrator, respectively. For ERG, OCT and weight data, the means for different age groups were compared using a Sidak's multiple comparisons test. In all experiments, *Bbs10^−/−^* mice were compared to littermate controls. Weight and OCT data were plotted as the mean and s.d. ERG was plotted as the mean and s.e.m. *P*-values for TTP in the swim assay were calculated using a unpaired two-tailed Student's *t*-test. All trial swim times were used to calculate the *P*-values, and data were plotted as the mean and s.e.m. All analyses were performed using an alpha value of 0.05.

## Supplementary Material

10.1242/dmm.049473_sup1 Supplementary information
