# Supplementary material for: Progressive retinal degeneration of rods and cones in a Bardet-Biedl syndrome type 10 mouse model
Source: Dis Model Mech. 2022 Sep 20;15(9):dmm049473. doi: 10.1242/dmm.049473 (PMC9536196; doi:10.1242/dmm.049473)
Supplement: Supplementary information [file dmm-15-049473-s1.pdf]

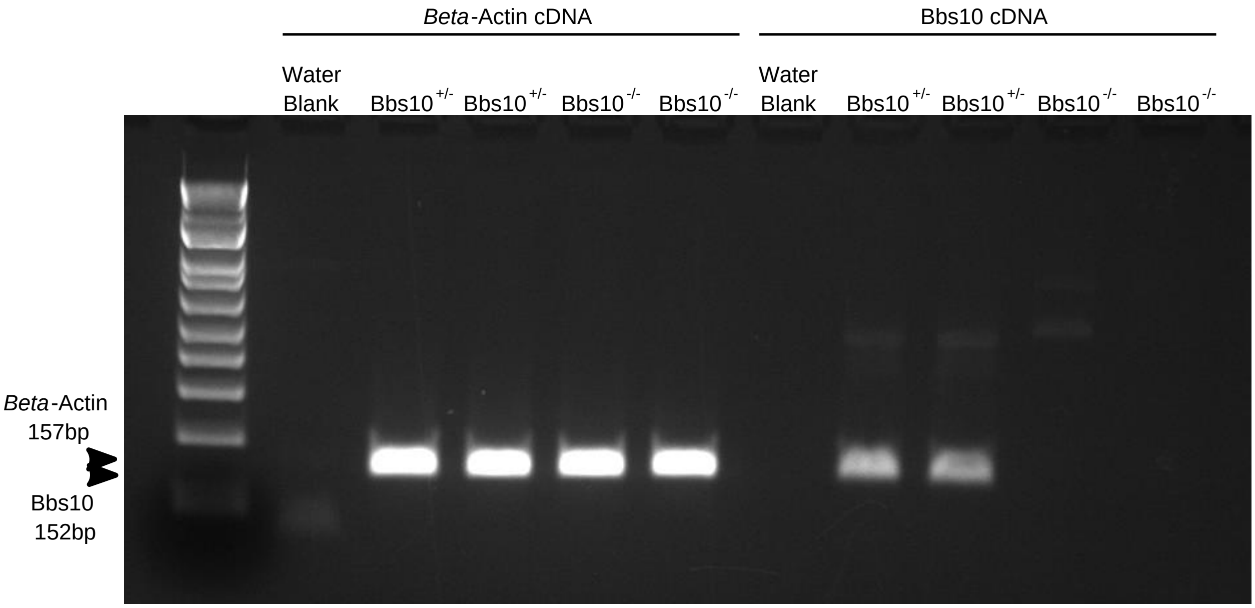

Fig. S1. *Bbs10*$^{-/-}$ mice lack the *Bbs10* mRNA. RT-PCR results for eye samples demonstrating that whereas both *Bbs10*$^{+/-}$ and *Bbs10*$^{-/-}$ produce robust amplification products for Beta-actin, only the *Bbs10*$^{+/-}$ mice produce detectable Bbs10 mRNA.

Disease Models & Mechanisms • Supplementary information

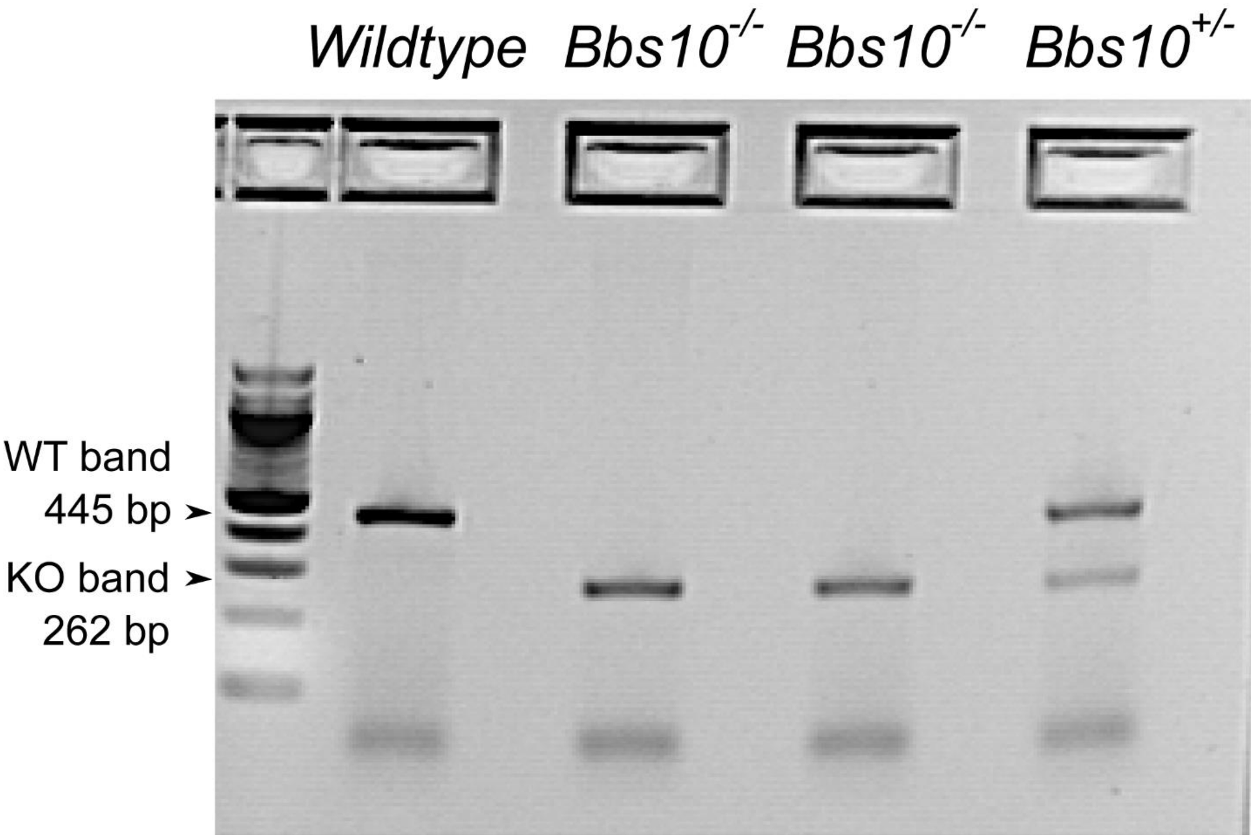

Fig. S2. In *Bbs10*$^{-/-}$ mice, which lack most of exons 1 and 2 of the *Bbs10* gene, the primer pair used produces a 260-bp band, whereas in wild-type mice they produce a 445-bp band. Heterozygous mice produce each product. The bottom band below 100bp is likely primer dimers.

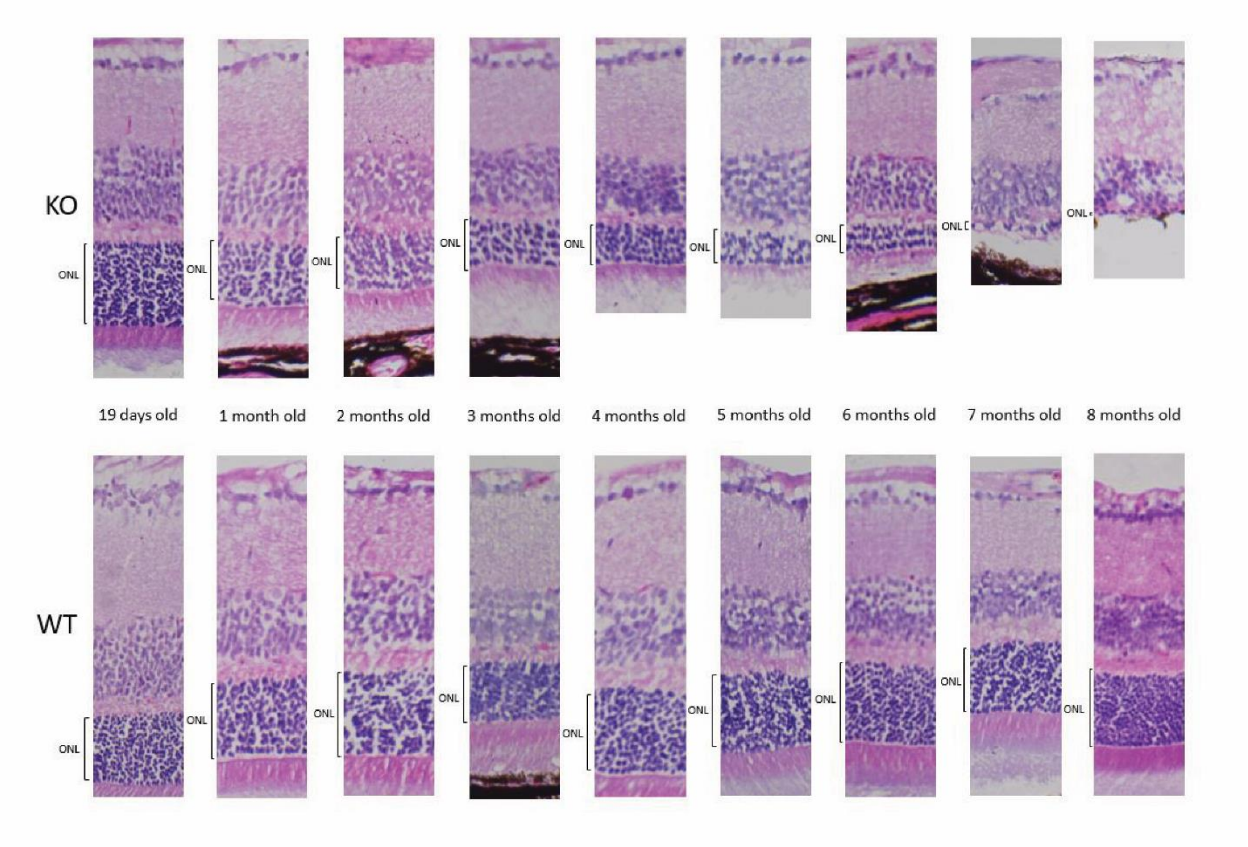

Fig. S3. ***Bbs10*$^{-/-}$ mice have a thinner outer nuclear layer (ONL) than control mice.** Hematoxylin and Eosin-stained sections of retinas from *Bbs10*$^{-/-}$ (KO) and wild-type (WT) mice at the specified ages. ONL is labeled in each sample.

Table S1. Primers used in genotyping and RT-PCR

Gene Name	Primer Sequence	Experiment	Product Size	Notes
mBact-qF	5' - TCGAGTCGCGTCCACC - 3'	RT-PCR	157 bp	
mBact-qR	5' - GGGAGCATCGTCGCCC - 3'	RT-PCR		
mBbs10-RT-F	5' - TGCTTAGCAGGGATGGAG - 3'	RT-PCR	152 bp	
mBbs10-RT-R	5' - TCCTCTGAGTAAATGGCAAAGA - 3'	RT-PCR		
mBbs10KO-WT-Fr	5' - CCCATGGTAAGTGGTCAATCAG - 3'	Genotypying PCR	445 bp	With Reverse (Rv) mBbs10KO
mBbs10KO-Mt-Fr	5' - TCAATGTATCTTATCATGTCTG - 3'	Genotypying PCR	260 bp	With Reverse (Rv) mBbs10KO
mBbs10KO-Rv	5' - TGGTCTGGTGGACTCAATGGAC - 3'	Genotypying PCR		
